# Bibliographical

**Published:** 1888-03

**Authors:** 


### BIBLIOGRAPHICAL.

IRREGULARITIES OF THE TEETH AND THEIR TREATMENT. By EUGENE S. TALBOT, M. D., D. D. S. With 152 illustrations. Philadelphia: P. Blakiston, Son & Co. 1888.

Dr. Talbot has, for some years, been known in the field of orthodontia as a man possessed of clear and well defined ideas respecting the origin of dental deformities, of extended experience and wide observation, as well as originality in devising appliances for the remedy of dental defects. This book is the summing up of the papers which he has at different times read before societies, and the results of his years of study. It forms a valuable addition to the literature of the subject, and no man will be enabled to boast of an acquaintance with it until he shall have mastered this book.

Yet the work is incomplete. The description of the appliances used is not sufficiently clear to enable the reader always to comprehend the method of their construction, nor are the principles involved sufficiently elaborated to constitute a full treatise. The author might plead that anyone who is sufficiently advanced in dental science to take up the study of irregularities should know the primary principles of mechanics, and it should be unnecessary to descend to detail. Yet the same argument would have deterred him from inserting an elementary chapter on anatomy, which contains the same cuts that are made to do duty for too many books of widely divergent character.

If the whole of the work had been devoted to an exposition of the methods and devices of Dr. Talbot himself, we think that it would have been more satisfactory, and the space would have been none too great. But when within its 160 pages the anatomy of the mouth is considered, and the systems of Patrick, Farrar, Guilford and others are included, it may readily be imagined that the presentation is too much condensed for entire perspicuity. Less than two pages, for instance, are devoted to Dr. Patrick's methods, which are barely sufficient to give them a brief notice. Dr. Angle is not mentioned at all, and hence we think it would have been better had the whole space been devoted to a consideration of the views and methods of Dr. Talbot himself. When the second edition shall be called for we shall look to see it considerably enlarged, or materially restricted in its scope.

PHOTOGRAPHIC ILLUSTRATIONS OF SKIN DISEASES. A complete work on Dermatology. By GEORGE HENRY FOX, A. M., M. D. Complete in twelve parts, with ninety illustrations from life, with hand colored plates. New York: E. B. Treat, 771 Broadway. 1888.

The second edition of this magnificent work is now in course of publication by E. B. Treat. When the first edition was issued, eight years ago, it marked an era in the illustration of pathological conditions, and established the value of photography, especially in delineating skin diseases. That edition met with such favor as works of the kind seldom receive, editions being published in France and Germany. This second edition is not a simple reproduction of the first, but is an entire remodeling of the work, with the text doubled in amount. The plates are from the original photographs, reproduced by Mr. Edward Bierstadt, by means of the unfading artotype process. The hand coloring has been done by Dr. Joseph Gaertner, a well-known medical artist, who was formerly a student under Von Hebra, in Vienna. The work is issued in the very highest style of the printing art, in the form of an atlas, the plates, on heavy cardboard ten by twelve inches in size, being inserted in the text.

From this imperfect description it may well be imagined that this is one of the most magnificent works on Dermatology ever issued.

It is something more than a medical book; it is a work of art as well. It is issued in twelve monthly parts, each part consisting of four plates, comprising from six to ten cases, at $2.00 per part. Parts I, II, III and IV are now ready, and may be obtained by addressing the publisher.

FEVER NURSING. Designed for the use of Professional and Other Nurses, and Especially as a Text Book for Nurses in Training. By J. C. WILSON, A. M., M. D. Philadelphia: J. B. Lippincott Company. 1888. Price, $1.00.

This is one of a series of hand-books issued from the well-known press of Lippincott, and intended as text books in the training schools for nurses, which have been established in connection with most of the great hospitals. It is made up from the lectures of the author before the class in the Philadelphia Hospital, and contains plain instructions in the duties of the nurse from the standpoint of the physician. But while it was primarily intended to supply a definite want, its usefulness will not by any means be restricted to that field. The practicing physician will find very much that will be instructive in one of his most important duties, the supervision of the care which his patient is to receive. Mothers, too, would do well to study it, for there are few whom it would not greatly benefit. In fact, all to whom the care of the sick is ever committed, whether professional or otherwise, should carefully note its recommendations and become familiar with its excellent teachings.

DENTAL METALLURGY. A Manual for the use of Dental Students. By CHAS. J. ESSIG, M. D., D. D. S. Second edition, revised. Philadelphia: The S. S. White Dental Manufacturing Co. 1888.

The first edition of this work was issued only about five years ago, and the fact that a second is so soon demanded speaks more in its favor than will the voice of the loudest of the critics. It has met the approval of dentists everywhere, and has been widely adopted as a text-book in dental schools. It is not a treatise—it is but a handbook—but it contains a great deal of information within a small compass.

The second edition is not merely a reprint of the first. The work has been revised, somewhat enlarged, and brought down to the present date, so that it is complete, so far as its scope will permit. We need not say that it is neatly printed and bound. The reputation of the publishers is a sufficient guarantee for that..

Beecher's Dental Directory of the United States. New York: Beecher & Co., 42 Third Avenue. 1888.

This handsome volume purports to be a complete directory of all the dentists of America. It contains, in addition, a complete list of all the leading dealers in and manufacturers of dental supplies, of dental colleges, dental publishers, etc. If its accuracy could be relied upon it is an invaluable publication, but we notice numerous errors which greatly mar its usefulness.

Six Hundred Medical Donts; or the Physician's Utility Enhanced. By Ferd. C. Valentine, M. D. New York: G. W. Dillingham. 1887.

We will add the Six Hundred and First "Dont," and it will be: Don't spend much time in the study of such silly trash as that which comprises the greater part of this book.

Die Ueberzahl und Unterzahl in den Zähnen des menschlichen Gebisses mit Einschluss der sogenannten Dentitio tertia.

(Excess and Deficiency in the number of teeth in the Human Race, including the so-called third Dentition.) By Prof. Dr. Busch. Berlin.

*Removal of Solid Uterine and Ovarian Tumors by Laparotomy;* with a report of nine cases. By Matthew D. Mann, A. M., M. D. Reprinted from *The American Journal of Obstetrics and Diseases of Women and Children.*

*Footprints of a Profession,* or Ethics in Materials and Methods. Address delivered before the Maine Dental Society at its twenty-second annual meeting. By Horatio C. Merriam, D. M. D.

*Operations for Mastoid Diseases. Statistical Report of 5,700 Cases of Ear Diseases.* Treatment of Chronic Suppurative Otitis Media. By Seth S. Bishop, M. D.

*Nasal Stenosis.* Its effect on the eye, ear, pharynx, larynx, voice and brain. By C. A. Bucklin, A. M., M. D. Reprinted from the *New York Medical Times.*

*A Series of Twenty-Five Laparotomies.* By Matthew D. Mann, A. M., M. D. Reprinted from *The Medical Press of Western New York.*

*Supra-Pubic Lithotomy.* A Historical Sketch. By Charles W. Dulles, M. D. Reprinted from the Trans. Medical Society of Pennsylvania for 1887.

